# Construction and Efficiency Estimation of a Nursing Human Resource Evaluation System in Integrated Medical‐Nursing Elderly Care Institutions Using Data Envelopment Analysis

**DOI:** 10.1155/jonm/6626385

**Published:** 2026-04-09

**Authors:** Mingxin He, Pengfei Cheng, Yan Liu, Ziwei Zhang, Yuhan Yang, Ka Yan Ho, Yanyan Li

**Affiliations:** ^1^ Department of Intensive Care Unit, Peking University Shenzhen Hospital, Shenzhen, 518036, Guangdong, China, pkuszh.com; ^2^ Faculty of Medicine, Macau University of Science and Technology, Taipa, 999078, Macau, China, must.edu.mo; ^3^ Neurology Department, Shenzhen Hospital (Futian) of Guangzhou University of Chinese Medicine, Shenzhen, 518034, Guangdong, China, shutcm.edu.cn; ^4^ School of Nursing, Hong Kong Polytechnic University, Kowloon, 999077, Hong Kong, China, polyu.edu.hk

**Keywords:** combination of medical care and elderly care, data envelopment analysis, evaluation of efficiency, health economics, nursing human resources

## Abstract

**Background:**

Efficient allocation of nursing human resources (NHR) is critical for optimizing care quality in integrated medical‐nursing elderly care institutions. However, standardized tools for assessing NHR efficiency remain underdeveloped.

**Objective:**

This study aimed to develop and validate a data envelopment analysis (DEA)–based evaluation system for nursing human resource efficiency in integrated elderly care institutions, with empirical application in clinical settings.

**Methods:**

Employing a cross‐sectional design, the research evaluated NHR efficiency in integrated medical‐nursing elderly care institutions. A preliminary indicator system was developed through comprehensive literature review and field investigations, followed by two rounds of Delphi consultations with 17 multidisciplinary experts specializing in nursing management, elderly care administration, integrated medical‐nursing operations, health economics, and public health. Expert reliability was determined by assessing response rates, authority levels (qualifications and experience), consensus (agreement rates), and coordination (Kendall’s *W* test). Based on this established indicator system, the DEA model was employed to evaluate the operational efficiency of 12 integrated medical‐nursing elderly care institutions in Hainan Province, China.

**Results:**

The constructed evaluation system featured a three‐level hierarchical structure totaling 68 indicators (9 first‐level, 19 second‐level, and 40 third‐level indicators). Two rounds of expert consultation demonstrated strong participation (response rates: 100% and 94.1%, respectively) and high reliability (authority coefficients: 0.88 and 0.92). Expert consensus was confirmed through statistical analysis (Kendall’s *W*: 0.471 and 0.348; average coefficient of variation: 0.16 and 0.12; all *p* < 0.001). Subsequent DEA implementation across 12 institutions revealed 5 were fully efficient (OE = TE = SE = 1.000), while the remaining 7 showed varying inefficiency patterns: 4 exhibited pure technical efficiency (TE = 1.000) with scale inefficiency (SE < 1.000), and 3 demonstrated both technical and scale inefficiency (TE < 1.000, SE < 1.000).

**Conclusion:**

The nursing human resource efficiency evaluation system developed in this study demonstrated strong validity through a rigorous Delphi expert consultation process, showing high expert engagement and authoritative consensus. The comprehensive three‐level indicator system exhibits well‐organized structure with strong specialty‐specific relevance for integrated medical‐nursing care settings. DEA application confirmed the system’s effectiveness in objectively evaluating nursing efficiency, supporting its practical utility for healthcare management in elderly care institutions.

## 1. Introduction

Aging is a significant concern encountered by the entire world [1–3]. Every country has implemented various types of elderly care models to tackle this challenge according to its national conditions. China, with a large population number, was ranked to have the highest number of aging populations around the world in 2024 [4, 5]. To address the increasingly complex medical and care needs of the aging population, the Chinese government has introduced the concept of medical‐elderly care integrated institutions, which represent an innovative service model combining medical treatment, elderly care, rehabilitation, and preventive health management within the same institution. This approach aims to provide older adults with comprehensive, continuous, and person‐centered services, thereby improving their quality of life and reducing the burden on families and the healthcare system [5]. Since the issuance of a series of policy documents, such as the Guiding Opinions on Promoting the Integration of Medical Care and Elderly Care Services by the State Council in 2015 [6], the development of such institutions has been strongly encouraged and supported nationwide. Compared with traditional elderly care facilities, medical‐elderly care integration emphasizes the seamless coordination between medical professionals and caregiving staff, ensuring timely diagnosis and treatment, effective rehabilitation, and day‐to‐day assistance in daily living activities [7]. Internationally, similar integrated care models have been implemented in countries such as Japan and the United Kingdom, where evidence suggests that coordinated medical and care services not only improve health outcomes among older adults but also enhance the efficiency of resource utilization [8, 9]. Drawing from these experiences, China’s model seeks to adapt integration principles to its unique demographic structure, healthcare system, and socio‐economic conditions [10].

Since its nationwide promotion in recent years, the implementation of medical‐elderly care‐integrated institutions in China has yielded positive outcomes in improving access to healthcare and long‐term care services for older adults [11]. However, multiple challenges have also emerged during the process, including uneven distribution of resources, insufficient service capacity in less‐developed regions, and lack of standardized management systems [12]. Among these, the mismatch between staffing levels and the actual care needs of elderly residents has been identified as a major obstacle to delivering high‐quality integrated services [12]. In particular, nursing staff play a pivotal role in ensuring continuity of care, coordinating medical and daily living assistance, and supporting rehabilitation and chronic disease management within these institutions [13, 14].

However, despite the vital role of nurses, a global nursing workforce crisis severely threatens the effective fulfillment of this role, especially within the long‐term care sector. This long‐standing crisis was critically accelerated by the COVID‐19 pandemic, which subjected frontline nurses to unprecedented physical and psychological stress, intense workloads, and professional burnout [15]. In the postpandemic era, far from abating, this crisis has intensified, compounded by multiple factors: a significant number of experienced nurses are leaving the profession due to burnout; the accelerated retirement of senior staff is leading to a loss of valuable clinical expertise; and new nursing graduates are often reluctant to enter the long‐term care field due to concerns about the work environment, compensation, and career prospects [16–18]. This predicament is not unique to China but is a global challenge. In the United States, research has revealed double‐digit postpandemic turnover rates among nurses, with staffing shortages reaching critical levels in long‐term care facilities, severely impacting care quality and financial sustainability [19]. In Europe, studies from countries like the United Kingdom, Portugal, and Greece have documented a worsening of nursing shortages, pointing to an aging workforce and an over‐reliance on international recruitment, which makes the labor market more vulnerable [20, 21]. In the Asia‐Pacific region, nations including South Korea, Japan, and China face similar struggles, where the high intensity of the work does not align with the levels of social recognition and remuneration, leading to a decline in the profession’s attractiveness to younger generations [22, 23]. This global contradiction between a supply‐side crisis (insufficient workforce) and growing demand‐side pressure (population aging) underscores the critical importance and urgency of optimizing the allocation and efficiency of existing nursing human resources (NHR).

Furthermore, previous studies have demonstrated that inadequate or inefficient allocation of NHR can directly affect care quality, patient satisfaction, and institutional sustainability [24, 25]. Therefore, in the context of the nursing human resource crisis, evaluating the efficiency of nursing human resource utilization is essential for optimizing workforce allocation, improving service quality, and promoting the sustainable development of medical‐elderly care integrated institutions [26]. However, the allocation and optimization of NHR have always been dynamic subjects that are constantly affected by the advanced elements of the era (such as theory, ideological trends, technology, etc.). In particular, when exploring how to optimize nursing human resource allocation, it is imperative to recognize that the field is undergoing a profound paradigm shift. Historically, the discourse on nurse staffing was long dominated by the “more is always better” paradigm. Pioneering research by scholars such as Aiken and Griffiths, for example, has provided substantial evidence linking higher nurse‐to‐patient ratios with lower patient mortality, fewer adverse events, and higher quality of care [27–29]. This paradigm has provided a robust empirical foundation for policies aimed at improving nurse staffing levels and ensuring patient safety worldwide. In recent years, however, this linear “more is better” mindset has faced increasing scrutiny and challenge, giving rise to the transitional paradigm of “more is not always better.” Researchers have found that simply increasing the number of nurses is not a panacea; its effectiveness is moderated by multiple factors, including skill mix, work experience, the organizational environment, technological support, and workflow processes [30]. Against this backdrop, a new generation of scholars, represented by Park, has critiqued the traditional paradigm and pioneered the new paradigm of “informed shared decision‐making” [31]. This new implementation strategy has multiple value potentials to cope with the growing nursing human resource shortage crisis. It not only balances the relationship between nursing quality, nursing staffing, and cost but also creates shared value among patients, nurses, and stakeholders through decisions based on sufficient evidence and information sharing so as to determine the optimal safe nursing staffing level. Especially for Asian countries dominated by collectivism that attach importance to safety responsibility and risk aversion, and economies with shrinking medical fiscal budgets caused by the economic downturn in the postpandemic era [32]. In addition, with the development of artificial intelligence, deep learning, and robotics, as well as the continuous integration of medicine and engineering, the solution to the allocation of nursing resources is also affected by other disciplines [33, 34]. To sum up, the evolution trajectory of the fundamental solution to the problem of nursing human resource allocation needs to be rooted in nursing philosophy and the paradigm innovation that fits the nursing management theory, as well as the continuous exploration and attempt at integrating multidisciplinary methods.

Any paradigm change and theoretical innovation in nursing staffing needs to be tested by practice, and Park’s Sweet Spot Theory is no exception. Its core idea is to seek a balance of multidimensional values (quality, cost, people), which sets a new, more scientific theoretical goal for the industry [32, 35]. But it also brings a new methodological challenge: How to empirically evaluate whether healthcare institutions are effectively achieving this complex balance in practice? Traditional efficiency assessment methods, such as simple nurse‐patient ratio or per capita service volume indicators, are no longer up to this task due to their single‐dimensional nature. They may mistakenly interpret the behavior of cutting costs or personnel as efficiency improvement, while completely ignoring the damage it may cause to the quality of care, which is exactly contrary to the core value advocated by the new paradigm. Addressing this challenge requires an empirical assessment tool that can integrate multiple dimensions of value. The data envelopment analysis (DEA) method in operations research provides a promising analytical framework, which can be used as an empirical tool to test and evaluate operational performance under the new paradigm [36]. DEA is a nonparametric mathematical technique that evaluates and compares the relative efficiency of a group of decision‐making units (DMUs)—such as hospitals, departments, wards, or individual service teams—by analyzing the relationship between their inputs (e.g., nurse staffing levels, working hours, bed numbers) and outputs (e.g., patient discharges, quality indicators, patient satisfaction) [37].

A growing body of literature has demonstrated that DEA is a robust and widely used quantitative method for assessing the efficiency of NHR utilization in healthcare settings [38, 39]. In nursing management research, DEA has been successfully applied to evaluate and benchmark the performance of clinical specialties, surgical wards, and intensive care units, thereby identifying inefficiencies and guiding resource reallocation. For example, Chen et al. [40] applied DEA to surgical nursing wards and demonstrated that objective efficiency scores can uncover hidden capacity and inform targeted staffing adjustments. Similarly, Bahrami et al. [41] highlighted the value of quantifying workload predictions in ICU settings, which could serve as measurable DEA inputs to optimize staffing patterns. However, its application in evaluating the efficiency of NHR within medical‐elderly care integrated institutions remains limited, particularly in the context of China. Although DEA has been increasingly applied to assess human resource efficiency in elderly care institutions, these applications have often been constrained by the absence of a standardized indicator system [42, 43]. Since DEA inherently depends on the selection of comparable input‐output measures, inconsistencies in indicator choice across studies not only limit the accuracy of efficiency estimation but also hinder cross‐institutional benchmarking [44]. This underscores the pressing need for a theoretically grounded and systematically constructed indicator framework tailored to the context of medical elderly care integration. Therefore, successful implementation of DEA in this field requires the development of a unified and standardized set of efficiency evaluation indicators to ensure the accuracy, comparability, and objectivity of the results [45]. At present, there is a paucity of scientific evidence and consensus regarding the specific indicators suitable for evaluating nursing human resource efficiency in medical elderly care integrated institutions.

Donabedian’s Structure–Process–Outcome (S–P–O) conceptual framework provides a solid theoretical foundation for addressing this challenge [46]. By integrating the Donabedian framework with the DEA method, our research aims to provide a critical, operational analytical tool for the “informed shared decision‐making” paradigm. The Donabedian model, widely recognized in healthcare quality research, divides evaluation dimensions into three interconnected domains: structure, process, and outcome. The conceptual clarity of this classification meets the needs of an efficiency‐oriented indicator system: Structure defines the foundational resources and organizational conditions; Process captures the organization and delivery of nursing work, and Outcome reflects residents’ health status, functional recovery, quality/safety metrics, and satisfaction. Because these three levels are conceptually distinct yet causally linked, they provide a scientific blueprint for indicator selection, helping to reduce structural bias and enhance cross‐institutional comparability​ [47]. The S–P–O framework also naturally aligns with the DEA method. In nonparametric efficiency studies, Donabedian‐type indicators are often mapped as inputs (primarily structure and some process workload indicators) and outputs (outcome and quality indicators), enabling a relative efficiency benchmarking against the best‐practice frontier. Indeed, care quality and human resource efficiency in nursing homes are an inseparable whole, with clinical care quality being a critical determinant of efficiency [48]. The work of Barsanti et al. [43] demonstrated that multidimensional care quality indicators are decisive factors in measuring nursing home efficiency outputs, thus recommending the inclusion of quality‐related variables in DEA‐based benchmarking studies. Methodological reviews also stress that incorporating quality/outcome variables into DEA models is essential to avoid potential biases in the “efficiency‐quality” trade‐off and to provide actionable signals for staffing optimization. In 2019, Tran et al. [42] conducted a systematic review of studies on nursing home efficiency measurement. Among the 39 studies analyzed, DEA and stochastic frontier analysis were the primary techniques used. Of these, 31 studies incorporated the multidimensionality of care quality and argued that efficiency assessments in long‐term care institutions must cover the three well‐validated and widely used quality dimensions of structure, process, and outcome as a minimum requirement to ensure the robustness and validity of the results. In summary, adopting the Donabedian framework not only provides a recognized and operational theoretical basis for indicator selection and classification but also seamlessly integrates with the input‐output model of DEA, supporting the use of structure and process indicators as inputs and outcome indicators as outputs for efficiency measurement and benchmarking. Thus, our research framework is no longer a simple efficiency measurement but constructs a multidimensional performance evaluation system that reflects care quality. The efficiency scores it produces are “value‐based efficiencies” that incorporate quality considerations, providing managers with a more profound and responsible basis for decision‐making.

Accordingly, the aims of this study are twofold: (i) To construct a scientific and standardized indicator system for evaluating the efficiency of NHR in medical‐elderly care integrated institutions, grounded in Donabedian’s conceptual framework and (ii) To apply DEA to measure and compare the efficiency of nursing human resource utilization across medical‐elderly care integrated institutions. By benchmarking input‐output performance, the DEA results will provide evidence‐based insights for nursing managers, assisting them in developing targeted management strategies aimed at achieving optimal allocation and rational utilization of NHR. Through a benchmarking analysis of input‐output performance, the DEA results from this study will provide nursing managers with a quantitative, quality‐inclusive, evidence‐based tool to support their judgment in the “informed shared decision‐making” process, with the ultimate goal of achieving intelligent, efficient, and high‐quality allocation and utilization of NHR.

## 2. Method

This study consists of two phases: (i) Construction of standardized efficiency indicators: A preliminary indicator system was developed through literature review and questionnaire survey, followed by a Delphi expert consultation to confirm its completeness, scientific validity, and feasibility and (ii) DEA‐based efficiency evaluation: Using the standardized indicators, DEA was employed to evaluate the efficiency of nursing resource allocation in integrated care institutions.

### 2.1. Construction of Standardized Efficiency Indicators

#### 2.1.1. Literature Review

**FIGURE 1:**
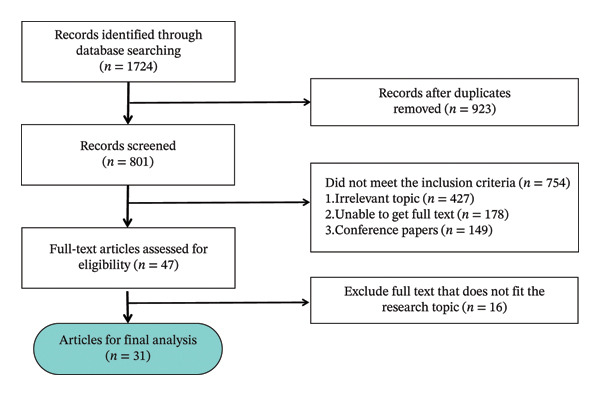
Results of literature screening.

Keywords such as “nursing/nurse,” “human resources/staffing,” “nursing home,” “integrated healthcare model,” “efficiency/technical efficiency,” “indicators,” and “data envelopment analysis” were used to search both Chinese and English databases, including CNKI, Wanfang, PubMed, Web of Science, Embase, Ovid, CINAHL, and Cochrane Library. The search period spanned from the inception of each database to January 2023. The inclusion criteria for the literature were as follows: (i) studies focusing on the evaluation of nursing human resource efficiency in elderly care institutions and (ii) publications in either Chinese or English. The exclusion criteria included (i) duplicate records or unavailable full texts and (ii) abstracts, conference papers, and research proposals. The literature screening process is summarized in Figure 1.

#### 2.1.2. Questionnaire Survey

This study employed a cross‐sectional survey methodology, during which a self‐administered questionnaire titled “Survey on Nursing Human Resource Allocation in Integrated Medical and Nursing Elderly Care Institutions” was developed based on a comprehensive literature review and in‐depth team discussions. The instrument encompassed key dimensions such as institutional infrastructure, patient admission profiles, medical department operations, and nursing staff allocation. Data collection was conducted from January to June 2023 among 12 institutions in Haikou and Sanya, Hainan Province, China, that met the predefined inclusion criteria—specifically (i) being an elderly care institution with integrated medical services and (ii) having been in operation for at least three years to ensure operational stability and model feasibility. Institutions that were temporarily non‐operational during the survey period were excluded.

#### 2.1.3. Delphi Method

The Delphi method is a structured communication technique originally developed by the RAND Corporation for systematic, interactive forecasting based on the input of a panel of experts [49]. Its key features include anonymity of participants, iterative rounds of questioning, controlled feedback (providing a statistical summary of the group’s previous responses), and the opportunity for participants to revise their judgments, thereby facilitating the convergence of opinions toward a reliable group consensus [50].

Based on a comprehensive literature review and a current‐state investigation, a preliminary set of evaluation indicators was developed, comprising 9 first‐level, 22 second‐level, and 45 third‐level indicators (76 items in total). A Delphi consultation questionnaire was subsequently drafted, structured as follows: (i) Introduction: This section outlines the research background, objectives, methodology, and instructions for completing the questionnaire. (ii) Evaluation indicators: Participants were asked to rate the importance of each indicator using a Likert 5‐point scale (1 = *completely unimportant*, 5 = *very important*). Space was provided for additional comments or suggestions for modification. (iii) Expert background and authority assessment: This section collected the experts’ demographic and professional data. To quantitatively assess the reliability of their judgments, each expert completed a self‐assessment on two key dimensions: Judgment basis (Ca) and familiarity with the content (Cs). For the Ca assessment, experts self‐rated the degree of influence for each of four potential judgment sources (practical experience, theoretical analysis, references to literature, and intuitive feeling) on their responses using a three‐level scale (“High,” “Medium,” “Low”), with corresponding pre‐assigned numerical weights: practical experience (high = 0.5, medium = 0.4, low = 0.3), theoretical analysis (high = 0.3, medium = 0.2, low = 0.1), references to literature (fixed weight of 0.1), and intuitive feeling (fixed weight of 0.1). The Ca score for each expert was calculated as the sum of the weights from all sources they considered influential, with a theoretical maximum of 1.0. For the Cs assessment, experts self‐rated their topic familiarity on a five‐point scale: very familiar (1.0), familiar (0.8), moderately familiar (0.6), unfamiliar (0.4), and very unfamiliar (0.2). The overall authority coefficient (Cr) for each expert was then derived using the formula: Cr = (Ca + Cs)/2. A Cr value > 0.8 is widely recognized as indicating a high level of expert authority.

##### 2.1.3.1. Selection of Experts

A total of 17 experts were selected for the Delphi consultation. The panel comprised professionals from various regions of China, including four from Hainan Province, three from Chongqing Municipality, two from Sichuan Province, two from Guangdong Province, and one each from Shanghai Municipality, Hebei Province, Hubei Province, Jilin Province, Jiangxi Province, and Tianjin Municipality. The selection criteria for experts were as follows: (i) Expertise in nursing management, management of elderly care institutions, management of integrated medical and nursing care institutions, health economics management, or public health; (ii) Holding a bachelor’s degree or higher; (iii) Possessing at least 10 years of relevant work experience or holding an associate senior professional title or above; and (iv) Willingness to participate in the study.

##### 2.1.3.2. Implementation of Expert Consultation

From July to December 2023, electronic consultation forms were distributed to solicit expert opinions via email or WeChat. The first round of consultation lasted for 14 days, and the second round for 7 days. After the first round, the indicator system was revised based on expert feedback and predefined item screening criteria to develop the second‐round questionnaire. This revised questionnaire was then resent to the experts. The consultation process was concluded when expert opinions showed a clear trend toward consensus. Subsequently, the research team further modified, adjusted, and refined the indicators accordingly.

### 2.2. DEA‐Based Efficiency Evaluation

#### 2.2.1. Model Selection

DEA is a nonparametric, linear programming‐based methodology used in operations research and econometrics to evaluate the relative efficiency of a set of comparable DMUs that utilize multiple inputs to produce multiple outputs [37]. In line with the research objective of objectively evaluating the efficiency of NHR within integrated medical and nursing care institutions for the elderly, the CCR and BCC models of DEA were adopted for efficiency estimation. The CCR model was used to evaluate the OE of the institutions, whereas the BCC model was applied to measure their TE.

#### 2.2.2. Principles for Selecting DEA Indicators

The selection of scientifically sound and reasonable evaluation indicators is fundamental to the application of DEA and serves as a core component of model construction. Therefore, this study adhered to the following principles in selecting efficiency indicators [51, 52]: (i) Relevance: Indicators should capture essential characteristics of the institutions; (ii) Scientific validity: Indicators must be empirically supported and theoretically justified; (iii) Data availability: Data for each indicator must be readily accessible within the evaluated units; (iv) Comprehensiveness: The indicator set should fully represent various aspects of efficiency utilization; (v) Correspondence: A logical relationship should exist between input and output indicators; (vi) Comparability: Indicators must be consistent and homogeneous across all DMUs; and (vii) Rule of thumb: The number of DMUs should be at least twice the total number of input and output indicators.

#### 2.2.3. Indicator Screening Process

In accordance with the thumb rule of DEA modeling, the total number of input and output indicators should not exceed half the number of DMUs, and the number of DMUs should be no less than the product of the number of input and output indicators [53, 54]. Based on the DMUs sample size in this study, it was determined that the total number of input and output indicators should not exceed five.

Due to the extensive scale of the initially constructed indicator system, a three‐stage screening process was implemented to refine the indicators. In the first stage, expert scoring values from the preliminary efficiency evaluation indicator database were used. Indicators were retained if they met the following criteria: a mean score ≥ 3.5, a coefficient of variation < 0.25, and a full‐score ratio > 70%. The second stage involved in‐depth discussion and analysis within the research team. Based on predefined inclusion criteria, the indicator set was further refined. Subsequently, data for these retained indicators were collected through on‐site field surveys conducted between January 1, 2022, and December 31, 2022, to ensure the validity and accuracy of the numerical data. In the third stage, Pearson correlation analysis was conducted on the indicators retained from the second round. Indicators exhibiting low correlation coefficients were eliminated. Among pairs of similar indicators with a correlation coefficient > 0.7 and statistical significance (*p* < 0.01 or *p* < 0.05), those with the five highest correlation coefficients were selected for retention.

#### 2.2.4. Model Data Collection

From January to March 2023, data corresponding to the indicators retained after the second round of screening were collected in paper‐based format. Following collection, on‐site verification was performed to ensure data completeness and accuracy.

#### 2.2.5. Model Calculation

From April to December 2023, an efficiency analysis was conducted using both the CCR and BCC models of DEA. The CCR model assumes constant returns to scale and measures overall technical efficiency, while the BCC model assumes variable returns to scale and decomposes efficiency into pure technical efficiency and scale efficiency. Input and output data from 12 DMUs that met the DEA inclusion criteria were incorporated into the models. The resulting efficiency scores ranged from 0 to 1.000. The effectiveness of the DEA approach was evaluated based on these outcomes. The mathematical formulations of the two models are presented as follows:

(1) CCRminθ−εemS−+esS+=VDε,∑j=1nXjλj+S−=θX0,∑j=1nYjλj−S+=Y0,λ≥000j=12,,⋯,n,S+≥,S−≥.

(2) BCCminθ−εemS−+esS+=VDε,∑j=1nXjλj+S−=θX0,∑j=1nYjλj−S+=Y0,∑j=1nλj=1, λj≥000j=12,,⋯,n,S+≥,S−≥.

### 2.3. Statistical Analysis

A database was established using Excel with dual data entry and verification. Data analysis was conducted using SPSS 26.0. (i) For quantitative data that followed a normal distribution, descriptive statistics were used for analysis. For categorical data, frequency and proportion were used for descriptive analysis. (ii) Delphi expert information data analysis: ① The response rate was used to represent the enthusiasm coefficient, with a response rate of > 70% indicating good quality [55, 56]; ② The authority coefficient was determined by the Ca coefficient and Cr coefficient, with Cr = (Ca + Cs)/2, and Cr ≥ 0.7 indicating that the research results are reliable [39]; ③ The degree of consensus was represented by the CV and Kendall’s *W*. CV is calculated as the standard deviation divided by the mean, with CV < 0.25 indicating good consensus. Kendall’s *W* ranges from 0 to 1, with *p* < 0.001 indicating high agreement and high approval of the scheme by experts [55]. (iii) The DEAP 2.1 software was used to analyze the data. The BCC model was used to evaluate the TE of the institutions, while the CCR model was used to evaluate the OE of the institutions. The efficiency values obtained ranged from 0 to 1.000.

## 3. Research Results

### 3.1. Development of Standardized Efficiency Indicators

This section primarily describes the preliminary development of relevant indicators through evidence‐based literature review and surveys, followed by the standardization, scientification, and refinement of these indicators using the Delphi method. The results of the Delphi expert consultation and the final constructed indicator system are presented below.

#### 3.1.1. Expert Demographics, Response Rate, and Kendall’s *W* of the Delphi Panel

##### 3.1.1.1. Expert Demographics

A total of 17 experts participated in the two‐round Delphi study. The panel comprised specialists in nursing management (*n* = 9), management of integrated institutions (*n* = 5), public health (*n* = 2), and health economics (*n* = 1). The majority (70.5%) possessed a postgraduate degree (master’s or higher), and 94.1% held senior professional titles. The mean age of the experts was 42.71 ± 3.12 years (range: 34–56), with an average work experience of 21.20 ± 4.32 years (range: 15–32).

##### 3.1.1.2. Response Rate

The response rate was 100% in the first round (17/17). In the second round, one expert was unavailable due to personal reasons, yielding a response rate of 94.1% (16/17). Both rates were well above the acceptable threshold of 70%.

##### 3.1.1.3. Expert Authority Coefficient

In the first round, the Cr was calculated as (0.94 + 0.85)/2 = 0.90. In the second round, Cr was (0.98 + 0.86)/2 = 0.92. Both values exceeded the satisfactory threshold of 0.70. Expert Consensus A significant consensus was achieved in both rounds. The Kendall’s *W* values were 0.471 (Round 1) and 0.348 (Round 2) (both *p* < 0.001). The corresponding mean coefficients of CV were 0.16 and 0.12. Detailed data are presented in Table 1.

**TABLE 1:** Analysis of consensus and significance test results from two rounds of expert consultation (*n* = 17).

Consultation round	Inquiry entry	Kendall’s *W*	Degrees of freedom	Mean CV	*χ* ^2^ value	*p* value
Round 1	76	0.471	75	0.16	600.13	< 0.001
Round 2	67	0.348	66	0.12	367.92	< 0.001

#### 3.1.2. Structure and Items of the Final Indicator Set

The constructed indicator system: Following the first round of the Delphi survey, the importance scores for each item ranged from 1.24 to 4.88, with SDs between 0.44 and 1.42. The full score ratios varied from 0% to 88%, and the coefficients of CVs ranged from 0.05 to 0.34. In the second round, all 17 invited experts participated, yielding 16 valid responses (a 94.1% response rate). After this round, the mean importance scores improved to a range of 3.56–4.90, with SDs from 0.25 to 1.21, full score ratios from 25% to 93%, and CV from 0.07 to 0.40. Based on the quantitative data and qualitative suggestions from the experts, the research team further discussed and refined the indicators. Consequently, a final indicator system for evaluating nursing human resource efficiency in integrated medical and elderly care institutions was established. The theoretical foundation for this system is Donabedian’s renowned S–P–O framework, which ensures a comprehensive assessment of foundational resources (Structure), care delivery activities (Process), and their ultimate effects (Outcome). The final system comprises a total of 68 items, categorized into 9 first‐level, 19 second‐level, and 40 third‐level indicators. Adhering to the S–P–O framework, these items are distributed as follows: 27 “Structure” indicators, 15 “Process” indicators, and 26 “Outcome” indicators. The detailed importance scores, CV, and full score rates for all final indicators are presented in Table 2.

**TABLE 2:** Evaluation indicators for nursing human resource efficiency of medical and elderly care integrated elderly care institutions.

Indicator and hierarchical items	S–P–O	Importance score ($\overset{¯}{x}$ + *s*)	Coefficient of variation	Full score rate (%)
1. Human resource input	S	4.88 ± 0.34	0.07	87.5
1.1. Number of personnel	S	4.77 ± 0.35	0.08	79.0
1.1.1. Number of licensed nurses	S	4.56 ± 0.51	0.11	56.3
1.1.2. Number of frontline licensed nurses	S	4.25 ± 0.86	0.20	43.8
1.1.3. Number of nursing interns	S	4.38 ± 0.72	0.16	50.0
1.1.4. Number of nursing trainees	S	4.24 ± 0.63	0.15	62.0
1.2. Nursing staff configuration structure	S	4.69 ± 0.48	0.10	68.8
1.2.1. Proportion of nurses with different education levels	S	4.56 ± 0.63	0.14	62.5
1.2.2. Proportion of nurses with different professional titles	S	4.56 ± 0.73	0.16	68.8
1.2.3. Proportion of nurses with different years of work experience	S	4.50 ± 0.52	0.11	50.0
1.2.4. Nurse‐to‐elderly ratio	S	4.50 ± 0.82	0.18	68.8
1.2.5. Proportion of nurses on sick or maternity leave	S	4.31 ± 0.79	0.18	50.0
2. Service input	S	4.76 ± 0.24	0.08	87.5
2.1. Daily care hours required for the elderly	S	4.44 ± 0.51	0.12	43.8
2.1.1. Care hours for elderly who are totally dependent	S	4.50 ± 0.89	0.20	68.8
2.1.2. Care hours for elderly who are partially dependent	S	4.75 ± 0.45	0.09	75.0
2.1.3. Care hours for elderly who are totally independent	S	4.56 ± 0.63	0.14	62.5
2.2. Rehabilitation programs provided	S	4.69 ± 0.48	0.10	68.8
2.2.1. Pulmonary rehabilitation exercise programs	S	4.50 ± 0.63	0.14	56.3
2.2.2. Stroke rehabilitation exercise programs	S	4.50 ± 0.52	0.11	50.0
2.2.3. Limb function rehabilitation exercise programs	S	4.31 ± 0.95	0.22	56.3
3. Financial input	S	4.74 ± 0.38	0.13	89.0
3.1. Annual salary income of nurses	S	4.53 ± 0.37	0.11	77.0
3.2. Training funds for education, science, and research	S	4.63 ± 0.50	0.11	62.5
3.2.1. Internal training funds	S	4.31 ± 0.70	0.16	43.8
3.2.2. External training funds	S	4.19 ± 0.54	0.13	25.0
4. Bed input	S	4.94 ± 0.25	0.05	93.8
5. Nursing service output	P	4.94 ± 0.25	0.05	93.8
5.1. Number of elderly admitted to the institution	P	4.50 ± 0.52	0.11	50.0
5.2. Bed occupancy rate of the institution	P	4.53 ± 0.45	0.13	62.5
5.3. Average length of stay for the elderly	P	4.72 ± 0.23	0.09	89.5
6. Nursing safety output	O	4.72 ± 0.24	0.06	86.0
6.1. Incidence of nursing adverse events	O	4.81 ± 0.40	0.08	81.3
6.1.1. Nurse medication errors	O	4.37 ± 0.49	0.16	56.3
6.1.2. Nurse failure to detect condition in time	O	4.69 ± 0.48	0.10	68.8
6.1.3. Unplanned tube removal	O	3.88 ± 1.02	0.26	37.5
6.1.4. Elderly wandering rate	O	4.63 ± 0.50	0.11	62.5
6.1.5. Elderly fall rate	O	4.52 ± 0.43	0.19	65.5
6.1.6. Elderly pressure ulcer rate	O	4.59 ± 0.44	0.17	70.8
6.2. Incidence of nursing occupational exposure	O	4.23 ± 0.37	0.12	79.0
7. Nursing quality output	P	4.79 ± 0.30	0.17	85.5
7.1. Nurse assessment pass rate	P	4.69 ± 0.48	0.10	68.8
7.1.1. Basic skill operation pass rate	P	4.75 ± 0.58	0.12	81.3
7.1.2. Basic theory assessment pass rate	P	4.56 ± 0.63	0.14	62.5
7.1.3. Elderly emergency plan assessment pass rate	P	4.63 ± 0.62	0.13	68.8
7.2. Nursing quality inspection pass rate	P	4.46 ± 0.37	0.15	56.3
7.2.1. 100% pass rate of medicine’s expiry date	P	4.52 ± 0.40	0.16	79.0
7.2.2. 100% pass rate of medical supplies expiry date	P	4.63 ± 0.50	0.11	62.5
7.2.3. 100% pass rate of resuscitation equipment in good standby condition	P	4.81 ± 0.40	0.08	81.3
7.2.4. Nursing documentation writing pass rate	P	4.56 ± 0.73	0.16	68.8
7.2.5. Health guidance and education pass rate	P	4.69 ± 0.48	0.10	68.8
7.3. Elderly treatment effectiveness rate	O	4.50 ± 0.73	0.16	62.5
7.4. Elderly transfer effectiveness rate	O	3.88 ± 0.81	0.21	25.0
8. Nursing “teaching, research, and innovation” output	O	4.38 ± 0.24	0.05	89.5
8.1. Talent cultivation	O	4.81 ± 0.54	0.11	87.5
8.1.1. Number of personnel with educational level upgrade	O	4.63 ± 0.72	0.16	75.0
8.1.2. Number of personnel with professional title upgrade	O	4.50 ± 0.63	0.14	56.3
8.1.3. Number of specialist nurses	O	3.56 ± 1.21	0.34	31.3
8.1.4. Number of interns and trainees taught	O	4.43 ± 0.36	0.12	75.0
8.2. Research output	O	4.81 ± 0.40	0.08	81.3
8.2.1. Number of invention patents and new technologies	O	4.69 ± 0.48	0.10	68.8
8.2.2. Number of articles published	O	4.63 ± 0.62	0.13	68.8
8.2.3. Number of research projects obtained	O	4.56 ± 0.63	0.14	62.5
9. Satisfaction	O	4.81 ± 0.40	0.08	83.3
9.1. Elderly satisfaction	O	4.63 ± 0.81	0.17	81.3
9.1.1. Satisfaction of the elderly themselves	O	4.56 ± 0.51	0.11	56.3
9.1.2. Satisfaction of elderly family members	O	4.75 ± 0.45	0.09	75.0
9.2. Nurse satisfaction	O	4.57 ± 0.41	0.13	82.0

*Note:* S–P–O classification based on Donabedian’s framework. S (Structure): resources and organizational characteristics. P (Process): activities of care delivery. O (Outcome): results of care.

### 3.2. Indicator Screening Process and Results for the DEA Model

#### 3.2.1. Indicator Screening Process

**FIGURE 2:**
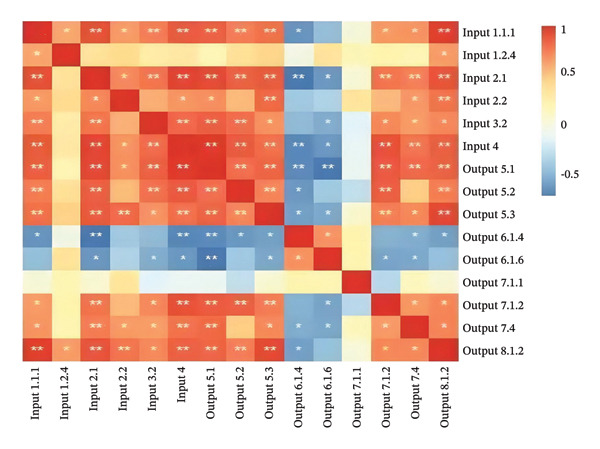
Heatmap of significance analysis of Pearson correlation coefficients for input and output indicators. Note: *p* < 0.01 (two‐tailed), the correlation is significant; *p* < 0.05 (two‐tailed), the correlation is significant.

In accordance with the thumb rule of DEA modeling, the total number of input and output indicators should not exceed half the number of DMUs, and the number of DMUs should be no less than the product of the number of input and output indicators. Given the sample size of DMUs in this study, the total number of input and output indicators was limited to five. Through three rounds of screening, five input‐output indicators with the highest correlation coefficients were ultimately selected. The detailed screening process is summarized in Table 3, and a heatmap of the Pearson correlation coefficients from the second round of screening is presented in Figure 2.

**TABLE 3:** The screening process of the DEA indicator model.

Screening steps	Screening criteria	Number of indicators	
		Input	Output
Step 1	Based on the scoring by experts in the consultation database, indicators with a mean score of 4, coefficient of variation < 0.25, and a full score rate of over 70% were included.	12	14
Step 2	The research team discussed the indicators, refined them according to the inclusion criteria, and subsequently performed Pearson correlation coefficient tests.	6	9
Step 3	Indicators with low correlation coefficients were eliminated based on the Pearson coefficient test analysis. For similar indicators, those with a correlation coefficient > 0.7 and *p* < 0.01 or < 0.05 were selected.	3	2

#### 3.2.2. Indicator Screening Results

Based on the “rule of thumb” for DEA modeling and constrained by the sample size of 12 DMUs, the total number of indicators that could be accommodated in the final model was limited to five. Accordingly, following the principle of “prioritizing statistical relevance while ensuring operational comparability,” the indicators initially selected through the Delphi method were discussed, after which Pearson correlation analysis was performed. This process led to the final determination of three input indicators (number of licensed nurses, daily care hours required for the elderly, and bed occupancy rate) and two output indicators (number of elderly admissions and average length of stay). These five indicators are statistically highly correlated and structurally coherent, enabling them to collectively capture the core characteristics of “resource‐to‐service” transformation efficiency. Furthermore, as all indicators are continuous, objective measures, they exhibit strong cross‐institutional comparability, thereby ensuring the discriminatory power and robustness of the DEA model under the constraint of a limited number of indicators. Although this screening process did not incorporate nursing quality‐related indicators—thus focusing primarily on operational efficiency assessment—the identified resource allocation issues provide an important structural optimization basis for enhancing service quality. Pearson correlation coefficients for these five indicators are presented in Table 4, and the clinical or operational significance of the selected indicators is detailed in Table 5.

**TABLE 4:** Pearson correlation coefficient values for input and output indicators.

Variable	Indicator 1.1.1	Indicator 2.1	Indicator 4	Indicator 5.1	Indicator 5.3
Indicator 1.1.1	1				
Indicator 2.1	0.909^∗∗^	1			
	< 0.001				
Indicator 4	0.867^∗∗^	0.946^∗∗^	1		
	< 0.001	< 0.001			
Indicator 5.1	0.839^∗∗^	0.926^∗∗^	0.974^∗∗^	1	
	0.001	< 0.001	< 0.001		
Indicator 5.3	0.798^∗∗^	0.860^∗∗^	0.785^∗∗^	0.822^∗∗^	1
	0.002	< 0.001	0.003	0.001	

*Note:* Correlation is significant at the 0.01 level.

^∗∗^ *p* < 0.01 (two‐tailed).

**TABLE 5:** Input and output indicators of the DEA model.

Indicator	Indicator attributes	Significance
Input 1.1.1. Number of licensed nurses	Human resource input	Annual number of employed nurses in the institution
Input 2.1. Daily care hours required for the elderly	Service input	Annual total care hours provided by nurses for the elderly with different self‐care abilities
Input 4. Bed input	Material input	Annual number of opened beds in the institution
Output 5.1. Number of elderly admitted to the institution	Nursing service output	Annual number of elderly admitted to the institution
Output 5.3. Average length of stay for the elderly	Nursing service output	Annual average length of stay for the elderly

*Note*: “Input” represents the input indicators, and “Output” represents the output indicators.

### 3.3. Efficiency Evaluation of 12 DMUs Based on DEA

The efficiency of 12 DMUs was evaluated using a DEA model, which analyzed TE, OE, SE, and slack variables (S^−^ and S+). Results indicated that 5 DMUs were DEA‐efficient (DMU1, 5, 7, 8, 10), while 7 were inefficient (DMU2–4, 6, 9, 11, 12). Among the inefficient DMUs, four showed SE values less than 1 but TE values equal to 1 (DMU3, 4, 6, 12), and three exhibited both SE and TE values less than 1 (DMU2, 9, 11). Regarding returns to scale, two inefficient DMUs (DMU2, 11) demonstrated increasing returns to scale, while five (DMU3, 4, 6, 9, 12) showed decreasing returns to scale. Detailed results are presented in Table 6.

**TABLE 6:** Evaluation results of nursing human resource configuration efficiency in 12 integrated medical and elderly care institutions.

DMU	OE	TE	SE	Returns to scale	S^−^	S^+^	Efficiency
1	1	1	1	—	0.000	0.000	Efficient
2	0.883	0.911	0.969	irs	96.146	7.087	Inefficient
3	0.858	1	0.858	drs	0.000	6.102	Inefficient
4	0.873	1	0.873	drs	0.000	0.000	Inefficient
5	1	1	1	—	0.000	0.000	Efficient
6	0.997	1	0.997	drs	3.133	0.000	Inefficient
7	1	1	1	—	0.000	0.000	Efficient
8	1	1	1	—	0.000	0.000	Efficient
9	0.835	0.857	0.974	drs	19.396	2.194	Inefficient
10	1	1	1	—	0.000	0.000	Efficient
11	0.850	0.893	0.952	irs	14.548	0.000	Inefficient
12	0.929	1	0.929	drs	58.190	6.217	Inefficient
Average	0.935	0.972	0.963				

*Note*: “—” indicates constant returns to scale.

Abbreviations: DMU, decision‐making unit; drs, decreasing returns to scale; irs, increasing returns to scale; OE, overall efficiency; SE, scale efficiency; TE, technical efficiency.

### 3.4. Evaluation Results of Slack Variables for Non‐DEA Efficient DMUs

For DMUs identified as DEA‐inefficient, corresponding improvement strategies should be formulated primarily based on their slack variable values. Slack variables reflect the degree of inefficiency in specific input or output indicators. S^−^ represents the amount of input that can be reduced to achieve the target efficiency, which is equivalent to the difference between the actual and target input values of non‐DEA‐efficient units. S^+^ indicates the amount of output that can be increased to attain the target efficiency, defined as the difference between the target and actual output values of non‐DEA‐efficient units. The slack value measures the gap between the actual value and the ideal (projected) value, where the projected value is obtained by subtracting S^−^ from the original input value or adding S^+^ to the original output value. The specific S^−^ and S^+^ values for each institution in this study are presented in Table 7.

**TABLE 7:** Analysis of slack variable values for non‐DEA efficient institutions.

DMU	S^−^			S^+^	
	Input 1.1.1	Input 2.1	Input 4	Output 5.1	Output 5.3
2	0.000	87.051	9.095	7.087	0.000
3	0.000	0.000	0.000	6.102	0.000
4	0.000	34.103	0.000	2.003	0.000
6	0.000	0.000	3.133	0.000	2.214
9	0.000	0.000	19.396	2.194	0.000
11	1.253	0.000	13.295	0.000	0.000
12	0.000	53.072	5.118	6.217	0.000

*Note*: “Input” represents the input indicators, and “Output” represents the output indicators.

## 4. Discussion

### 4.1. Core Findings and Theoretical Dialog

#### 4.1.1. Development and Validation of the S–P–O‐Based Indicator System

The primary contributions of this study are twofold. First, this study devised a comprehensive, theory‐driven evaluation system for NHR in integrated medical‐nursing care institutions. To address the notable lack of empirical tools, a 68‐indicator system was constructed, grounded in Donabedian’s S–P–O framework via a robust two‐round Delphi process. The system’s credibility is underscored by the rigor of the expert consultation [57–59]. The panel demonstrated strong representativeness, high engagement (response rates: 100% and 94.1%), and authoritative expertise (authority coefficients > 0.8), collectively ensuring rigorous indicator selection and minimized bias. A key methodological finding was the evolution of expert consensus. The significant Kendall’s *W* values in both rounds (0.471 and 0.348, *p* < 0.001) confirm strong agreement, while the decrease in the second round reflects the Delphi process’s capacity for refinement. Importantly, the parallel decrease in the mean coefficient of CV from 0.16 in Round 1 to 0.12 in Round 2 provides further quantitative evidence of this consensus convergence. In Delphi studies, a lower CV indicates reduced dispersion in expert ratings, representing a higher degree of agreement on the specific value or importance of each item. This decline signifies that after considering the anonymized group feedback, experts’ judgments converged toward a common viewpoint. The simultaneous improvement in both statistical measures—refined ranking consistency captured by Kendall’s *W* and increased concentration in rating scores reflected in a lower CV—demonstrates a successful transition from broad structural agreement to a nuanced, detailed consensus on operational specifics. Iterative feedback transformed the initial broad structural consensus into a critical, nuanced refinement of indicator wording and applicability. Therefore, this rigorous process culminates in a comprehensive, clear, and readily applicable evaluation system. This specialized tool fills a critical gap by providing a standardized, evidence‐based foundation to inform both management practice and future research in integrated care settings.

#### 4.1.2. Key Findings From DEA Efficiency Estimation

In addition, leveraging this system, the study proceeded to conduct an empirical efficiency estimation of NHR across 12 representative institutions by employing DEA. The principal finding from this estimation is that the overall efficiency among the sampled institutions is generally low, with only a minority (5 out of 12) operating at the efficiency frontier (i.e., being DEA‐efficient). A deeper analysis reveals that the root causes of this inefficiency are varied: Some institutions are constrained by poor PTE, indicating suboptimal management and operational processes, while others are hindered by inadequate SE. Critically, the majority of inefficient institutions (5 out of 7) were found to be operating under DRS. This strongly suggests that their level of resource input, such as nursing staff and beds, may have already exceeded the optimal scale, meaning that further increases in inputs would likely fail to improve efficiency and could instead lead to greater resource redundancy. These findings collectively argue against a one‐size‐fits‐all approach, highlighting instead the necessity for tailored, evidence‐based strategies that address the specific technical or scale‐related inefficiencies of each institution.

#### 4.1.3. Theoretical Dialog: Supporting a Paradigm Shift From “More is Better” to “Informed Optimization”

For a long time, many studies have found that the scientific and rational allocation and utilization of NHR within medical and nursing institutions not only helps to offer more nursing service programs but also reduces the incidence of accidental events among the elderly [60]. Our findings, however, illuminate the complexities inherent in the practical application. The results from our DEA reveal a critical disjuncture: a significant number of institutions with high, or even above‐average, levels of nursing staff input still exhibited low efficiency scores. This indicates the presence of resource redundancy or suboptimal allocation structures, empirically confirming the theory‐practice gap that the “more is better” paradigm faces under real‐world cost and resource constraints. More fundamentally, our study exposes the core of the issue from an efficiency perspective: a singular focus on increasing the quantity of nursing staff, while neglecting the efficiency of their allocation, may lead not to optimized value creation but to the inefficient dissipation of resources. Consequently, our results lend strong support to an emerging paradigm shift centered on the principle that “more is not always better” [32]. This intellectual movement, spearheaded by Park, challenges traditional staffing models by arguing that strategies aimed solely at increasing nurse numbers often become “unimplementable” because they fail to specify an optimal balance point between cost and quality [31, 35]. In response, Park has pioneered a new paradigm of “informed shared decision‐making,” which seeks to identify a “sweet spot,” a scientifically grounded and feasible range for staffing that balances cost‐effectiveness with efficiency, to guide policy and practice [32]. It is worth noting that our study is a positive response to this new paradigm advocated by Park and a concrete practice at the methodological level, but there are attribute differences in methods and theories. To be specific, DEA is a retrospective, empirical diagnostic tool. It leverages historical input‐output data to conduct relative efficiency benchmarking across a cohort of homogenous institutions. Its primary function is diagnostic: to identify best‐practice efficiency frontiers and to quantify resource slack in underperforming units [40]. DEA is therefore a managerial tool that helps administrators optimize operations under existing conditions, answering the questions, “How are we performing now?” and “From whom can we learn to improve?” In contrast, Park’s Sweet Spot Theory is a prospective, theory‐driven, and normative approach [31]. Rooted in nursing philosophy and theory, its objective is to identify and predict an optimal range for safe nurse staffing. This “sweet spot” represents a normative ideal—a golden equilibrium of cost‐effectiveness and cost‐efficiency [32]. Park’s theory is thus designed to furnish policymakers with a predictive, macro‐level target, answering the question, “What should our system‐wide staffing goal be to achieve an optimal state?” In summary, our DEA study and Park’s sweet spot theory play highly complementary rather than competitive roles under the common paradigm that more is not always better. Park’s theory provides a prospective, system‐level theoretical goal for “optimal allocation.” In contrast, our DEA study provides a retrospective, institution‐level empirical management tool to assess the distance of individual institutions from the “efficiency frontier” and to indicate concrete improvement paths for them toward optimization. Going further, this study provides an empirical basis for implementing the more advanced decision‐making framework advocated by Park.

### 4.2. From Diagnosis to Action: Managerial Implications and an Optimization Roadmap

The value of this study transcends a mere efficiency ranking by leveraging slack variable analysis to formulate a multifaceted and targeted strategic roadmap for inefficient institutions. This methodology substantiates the “more is not always better” paradigm within complex systems like elderly care, translating abstract efficiency scores into concrete managerial actions. On the input side, for instance, the minor slack in nurse input (Input 1.1.1) at DMUs11 does not simplistically suggest overstaffing but rather points to a more nuanced issue of skill‐mix inefficiency, where highly skilled professionals may be mired in tasks below their qualification, mandating workflow and role optimization over headcount reduction [37, 61]. In stark contrast, the substantial slack in support staff (Input 2.1) across several DMUs epitomizes how bloated administrative structures fail to correlate with higher productivity; the remedy for technically inefficient units is internal process re‐engineering via Lean principles, while for scale‐inefficient units, exploring shared‐service models to achieve economies of scale is advisable [38]. Similarly, widespread slack in fixed assets (Input 4) signifies underutilized capital, which should be addressed either by enhancing service visibility to drive demand or by strategic service diversification to repurpose idle assets, depending on the unit’s efficiency type [62]. Concurrently, the output analysis is equally prescriptive. Shortfalls in daily care volume (Output 5.1) reveal latent productive capacity constrained by operational friction, which can be unlocked by implementing standardized care pathways. Meanwhile, a deficit in quality‐of‐life interventions (Output 5.3), as seen in DMU6, highlights a missed opportunity to enhance the value proposition and market differentiation, necessitating a strategic realignment toward high‐value services [63]. In essence, this granular, indicator‐specific approach enables remedies—ranging from process re‐engineering and structural reorganization to strategic repositioning—to be precisely tailored to the nature and locus of each institution’s identified shortcomings, providing a truly actionable optimization plan [64].

### 4.3. Broader Implications for Nursing Quality and Staff Well‐Being

Beyond numerical efficiency scores, our findings also carry significant organizational implications. Higher efficiency not only reflects optimal utilization of nursing resources but may also contribute to improved care quality, fewer missed nursing activities, and enhanced patient satisfaction, as supported by prior nursing management literature [65]. Conversely, excessively high efficiency scores may risk overstretching the nursing workforce, potentially undermining staff satisfaction, increasing burnout, and compromising care quality [66]. This highlights the need to balance efficiency gains with quality and workforce well‐being, a point critically raised for operationalization. To quantitatively and qualitatively assess this trade‐off, future research should adopt a multifaceted analytical framework. First, the relationship can be examined by correlating DEA efficiency scores with a set of specific, measurable indicators. For care quality, these would include key elderly health outcomes such as fall rates, pressure ulcer incidence, and unplanned hospital readmission rates, supplemented by resident and family satisfaction surveys. For nursing workforce well‐being, crucial metrics include staff turnover rates, absenteeism, and validated psychometric scales like the Maslach Burnout Inventory to measure burnout. Methodologically, a two‐stage analysis provides a robust approach to investigate this trade‐off. In the first stage, DEA is used to calculate efficiency scores, as done in our study. In the second stage, these scores are used as an independent variable in a regression model (e.g., Tobit or OLS regression) to predict the aforementioned quality and well‐being indicators. Critically, by including the squared term of the efficiency score (Efficiency^2^) in the regression, we can test for a nonlinear, inverted U‐shaped relationship. A significant negative coefficient on this squared term would provide empirical evidence of a tipping point, beyond which further increases in efficiency lead to diminishing returns or even negative impacts on care quality and nurse satisfaction. This would quantitatively define the boundary between beneficial efficiency and detrimental overstretching. Therefore, the interpretation of DEA results should be situated within this broader analytical framework, encompassing care quality, staff satisfaction, and resource utilization. Doing so ensures that improvements in efficiency are meaningful and sustainable in practice, supporting the dual goals of delivering high‐quality, person‐centered care and maintaining a motivated, resilient nursing workforce.

### 4.4. Study Limitations and Contextual Considerations

At the same time, this study also acknowledges several limitations, which can be categorized into three main areas: the conceptual scope of the indicators, sample representativeness, and the international applicability of the model. First, the conceptual and methodological scope of the efficiency evaluation is constrained by the selected indicators. While Donabedian’s S–P–O model is a well‐established conceptual framework for quality assessment, it is not a nursing‐specific theory. Consequently, the diversification of outcome measures may still overlook broader aspects of nurse well‐being. To address this, future research should strive to incorporate a more diverse set of outcome‐oriented metrics. This expansion should move beyond operational data to enable a comprehensive performance assessment: starting with staff well‐being, evaluated through nurse‐related outcomes such as burnout levels, job satisfaction, turnover rates, and occurrences of missed nursing care; simultaneously assessing direct impact on patients by tracking quality of life, functional recovery progress, readmission and infection rates, and satisfaction scores from patients and their families; and further refining this holistic perspective through organizational outcomes that reflect system health, such as continuity of care, effectiveness of interdisciplinary collaboration, and measurable professional development achievements. Second, limitations related to sample size and research timeline. The indicator system was constructed based on field research conducted in a single city, resulting in a DEA analysis that included only 12 DMUs. The small, geographically concentrated sample not only restricted the number of indicators that could be incorporated but also confined the analysis to a cross‐sectional perspective, which may affect the external validity of the findings. However, this limitation also reveals a practical strength of the approach: even in settings with limited sample sizes or data resources—such as small or medium‐sized cities or specific regions—the model remains capable of facilitating effective intra‐regional horizontal comparisons and efficiency measurements, providing a viable analytical tool for localized management decision‐making. Furthermore, constrained by the research timeline, we were unable to conduct longitudinal follow‐up to assess the effects of the proposed optimizations, despite having provided the evaluation results and recommendations to participating institutions. This longitudinal verification has been explicitly prioritized in our subsequent research agenda. Third, the applicability of the constructed indicator system in international contexts has not yet been validated. Although the development process drew upon and incorporated common indicators from international studies, the final system was primarily tailored to the specific context and policy environment of China’s healthcare system. Therefore, direct application to other countries or regions may require adaptation of certain specific indicators. Nevertheless, the theoretically driven methodology, rigorous Delphi expert consultation process, and transparent multistage screening procedure employed in this study collectively form a logical, rigorous, and replicable scientific framework. This framework itself provides a feasible and efficient reference pathway for other researchers to systematically develop localized nursing human resource efficiency evaluation tools according to their respective national contexts.

### 4.5. Recommendations for Future Inquiry and Methodological Refinement

The findings and limitations of this study collectively provide clear direction for future research. Subsequent work can advance along two pathways. On one hand, by expanding the geographical and typological coverage of the sample and employing super‐efficiency DEA models, horizontal efficiency comparisons and longitudinal cross‐period efficiency analyses can be conducted on a broader scale. This approach would facilitate the dynamic tracking of efficiency evolution trends and provide empirical validation for the institution‐specific optimization strategies based on slack variables proposed in the earlier phase of this research. On the other hand, a more systematic three‐stage methodological framework of “consensus screening‐scientific weighting‐statistical optimization” can be established. Specifically, building upon the initial screening based on expert rating consensus (e.g., mean score, coefficient of variation, and full‐score rate), structured weighting methods such as AHP or entropy weighting can be integrated to scientifically quantify the relative importance of indicators, followed by the application of Pearson correlation analysis for statistical screening and dimensionality reduction. This integrated strategy systematically combines qualitative consensus, theoretical weighting, and data‐driven characteristics, optimizing the indicator structure while maximizing the retention of critical information, thereby significantly enhancing the scientific rigor and generalizability of the evaluation system. Overall, these pathways not only contribute to deepening methodological inquiry in this field but also offer a systematic and transferable approach for developing context‐adapted evaluation tools across diverse healthcare settings.

## 5. Conclusion

This study, building upon the previously established evaluation indicator system for nursing human resource efficiency in integrated medical and elderly care institutions, employed the DEA–BCC and CCR models to assess efficiency. The results indicate that non‐DEA efficient institutions exhibit redundancy in nursing human resource input and/or insufficient output. Based on the values of slack variables, institution managers can develop tailored optimization strategies according to their specific circumstances and also refer to DEA‐efficient DMUs to enhance the utilization of nursing human resource efficiency and achieve sustainable development. In summary, by situating DEA analysis within Donabedian’s conceptual framework and comparing our findings with existing applications in hospital nursing units, this study demonstrates the feasibility and value of efficiency evaluation for NHR in medical‐elderly care integrated institutions. The insights generated can inform evidence‐based management strategies, strengthen care delivery, and promote sustainable workforce practices in the context of population aging.

## 6. Implications for Nursing Management

The results of this study offer several implications for nursing management. First, DEA can be employed as a diagnostic tool to identify inefficiencies in staffing patterns and resource allocation, providing an evidence base for strategic workforce planning in medical‐elderly care integrated institutions. Second, the establishment of standardized efficiency indicators, guided by Donabedian’s framework, enables managers to conduct transparent benchmarking across institutions and over time, fostering a culture of continuous improvement. Finally, efficiency evaluation results can support the design of targeted professional development and training programs, ensuring that staff skills align with institutional needs while improving overall workforce adaptability. Together, these implications highlight the critical role of nursing leadership in translating efficiency metrics into meaningful, actionable strategies that advance the goals of integrated elderly care.

## Author Contributions

All listed authors have made substantial intellectual contributions to this research. The specific contributions of each author are detailed below using the CRediT (Contributor Roles Taxonomy) framework. Individual author contributions: Mingxin He and Pengfei Cheng: conceptualization, methodology, investigation, formal analysis, visualization, writing–original draft, and writing–review and editing. Yan Liu, Ziwei Zhang, and Yuhan Yang: data curation, formal analysis, resources, and writing–review and editing. Ka Yan Ho: writing–review and editing and supervision. Yanyan Li: conceptualization, funding acquisition, project administration, supervision, validation, and writing–review and editing.

## Funding

The present study was supported by the Research Project on Nursing Innovation and Development of Guangdong Nursing Society in 2024 (GDHLYJYM 202426); Research Project of Guangdong Nurses Association (gdshsxh2024zd04); Teaching Reform and Research Project of Shantou University School of Medicine in 2024 (24JXGG53); the Internal Research Special Project of Shenzhen Hospital (Futian) of Guangzhou University of Chinese Medicine (GZYSY2024025); Scientific Research Projects of Shenzhen Health Economics Association (2025261, 2025165, 2025242, 2025134).

## Disclosure

All authors have approved the final version of the manuscript for submission.

## Ethics Statement

This study was reviewed and approved by the Ethics Committee of The Hainan Medical University [HYLL scientific research (No. 2022‐009)]. Written informed consent was obtained from all individual participants prior to their inclusion in the study.

## Consent

Please see the ethics statement.

## Conflicts of Interest

The authors declare no conflicts of interest.

## Data Availability

The data that support the findings of this study are available from the corresponding author upon reasonable request.
